# Three novel structural phenomena in the cellular ontogeny of *Oenococcus oeni* from northern China

**DOI:** 10.1038/s41598-017-09685-1

**Published:** 2017-09-12

**Authors:** Yun Wang, Shuwen Liu, Jing Su, Yu Zhang, Jing Li, Yin-Qiang Sui, Ying-Ying Li, Hua Wang, Hua Li

**Affiliations:** 1 College of Enology, Northwest A&F University, Shaanxi, 712100 China; 2 Shaanxi Engineering Research Center for Viti-Viniculture, Shaanxi, 712100 China; 3 Heyang Experimental and Demonstrational Stations for Grape, Shaanxi, 715300 China; 4 College of Food Science and Engineering, Northwest A&F University, Shaanxi, 712100 China

## Abstract

Stress resistance and growth are important aspects to consider when engineering *Oenococcus oeni* strains for winemaking. We identified 3 previously unreported structural phenomena in the cell ontogeny of *O*. *oeni* sampled in northern China. We show that budding and binary fission (BBF) occur simultaneously in the growth process; that a novel ‘pomegranate-shaped structure’ (PSS) occurs mainly in the stationary and death phases; and that symbiosis and cyclical phenomena (SCP) occur throughout the various cell growth phases. These observations add to the current knowledge of the cell growth process of *O*. *oeni*. BBF, PSS, and SCP sufficiently describe the characteristics of the cellular ontogeny of *O*. *oeni*. We highlight a newly identified structure that explains the complex cell growth process. These findings will help understand the growth and development of *O*. *oeni*, supplementing the knowledge base of the established phases and providing new perspectives into its complex growth patterns.

## Introduction

Over a century ago, Pasteur recognised that lactic acid bacteria (LAB) carry out malolactic fermentation (MLF). The underlying bacterium was subsequently identified and named *Leuconostoc oenos* (i.e. displaying a chain shape) by Garvie^1^. In 1995, it was renamed by Dicks as *Oenococcus oeni* (i.e. chain and spherical shape)^2^. Current research on wine LAB has progressed to the molecular level^3^. LAB are responsible for the MLF that follows alcoholic fermentation by yeasts in winemaking, which improves wine stability and quality. MLF usually occurs either spontaneously or after inoculation with selected strains after the alcoholic fermentation step. One of the LAB responsible for MLF, *O*. *oeni*, develops in physicochemically harsh conditions, for example, under high temperatures and pH, which can affect the success of MLF. Therefore, developing stress-resistant *O*. *oeni* strains is imperative for their use in the winemaking industry.

**Figure 1:**
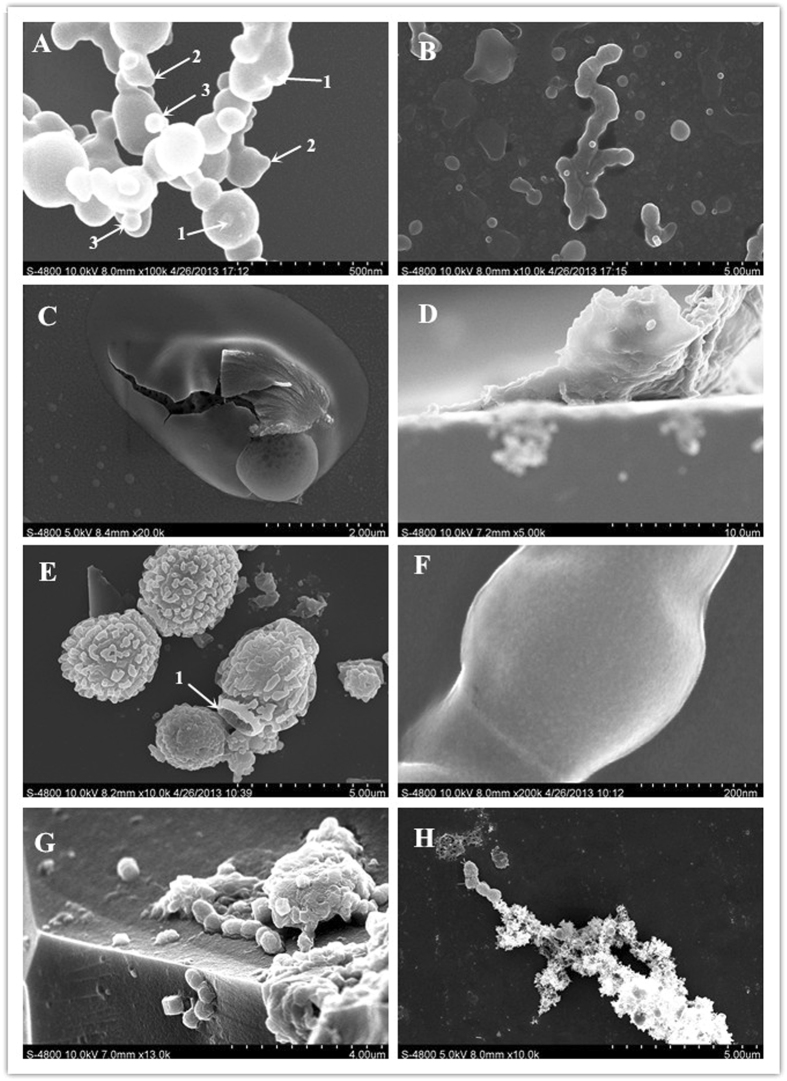
Unusual phenomena noted in the initial stages of the experiment. (**A,B**) Budding division and binary fission occurring simultaneously during the cell growth period (**B**, budding division in chains). (**C,D**) Pomegranate-shaped structure (PSS) in the later period and PSS fragments. (**E,F**) Two layers of cell chains and the sleeve membrane of the cells. (**G,H**) Co-occurrence of chains and PSS fragments (scaffolds) of various sizes during the different growth periods of the strain.

Various strains have been sampled from northern China, the major wine-producing belt of China^4^. However, some unusual phenomena were noted during the initial bacterial examination using field-emission scanning electron microscopy (FESEM), such as simultaneous budding and binary fission (BBF) in the growth phase (Fig. 1A and B) and a pomegranate-shaped structure (PSS) in the later period (Fig. 1C and D). Because published studies on PSS in bacteria and fungi are not available, we referred to the individual development of the actual pomegranate fruit to compare the mechanism of pomegranate fruit growth and the apparently similar changes in *O*. *oeni* cells during their growth. Clear observation of 2 layers of cell chains and sleeve membranes (Fig. 1E and F) and their varying sizes during the different growth periods of the experimental strain (Fig. 1G and H) suggested that the strains were in a state of ‘symbiosis’—a rather controversial finding—because the budding phenomenon has never been observed in LAB (which belong to the bacterial phylum *Firmicutes*). However, yeasts (fungi), *Proteobacteria*, planktonic bacteria, and *Ruminococcus* (phylum *Actinobacteria*) have been found to demonstrate the budding phenomenon.

In addition, we modified 4 sample preparation methods of FESEM and 3 of transmission electron microscopy (TEM). The sample preparation time was shortened from 24 h to 2 h to maintain the natural shape of the strains as much as possible, while allowing sample collection from different growth periods to observe and analyse the performance and variation of the different strains. In total, 1759 FESEM and 436 TEM images were observed, and measurements were obtained from 1037 images (Table 1). This provided a comprehensive understanding of these bacteria. Here, we introduce 3 new structural phenomena that complete the known phases of the *O*. *oeni* growth period and highlight new perspectives on the complexity of this process.

**Table 1:** Measurements and calculations obtained from the images.

Measured (nm)	0–100	101–200	201–300	301–400	401–500	501–600	601–700	701–1000	1001–2000	2001–5000	>5000
BBF (No.)	27	30	20	75	59	22	7	8	20	20	3
% in 201–600 nm	60.50%										
PSS (No.)	86	32	67	168	201	68	19	33	48	42	9
% in 201–600 nm	65.20%										
Spherical (No.)	91	71	49	57	48	35	14	36	36	8	0
% in 100–600 nm	70.60%										
Chains (No.)	3	13	42	496	762	253	32	10	6	5	0
% in 301–600 nm	93.20%										

## Results and Discussion

### Simultaneous occurrence of budding and binary fission

**Figure 2:**
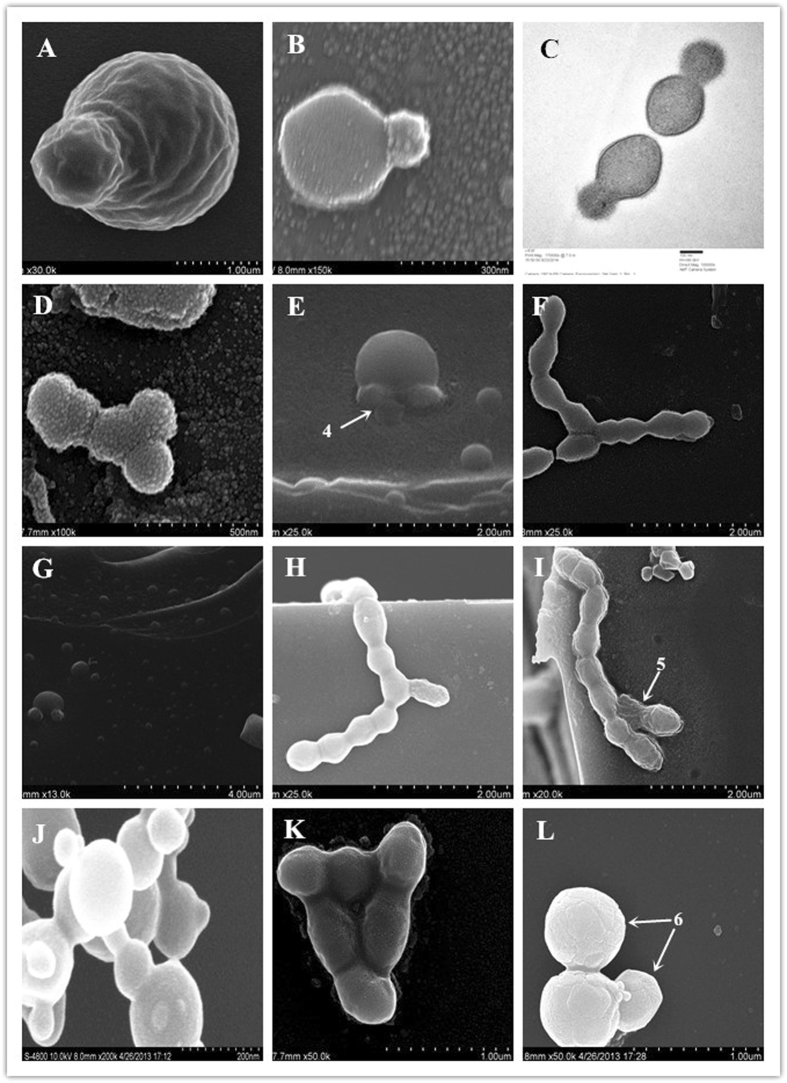
Images of the BBF phenomenon. (**A–C**) Outward protrusion in the centre of the surface of spherical cells, forming 1 or several small protrusions (**C**, TEM image). (**D,F** and **G**) Outward protrusion of the lateral surface of the cell chains (in the normal position of binary fission), forming 1 or several small protrusions. (**H–J**) Outward protrusion of the lateral surface of the cell chains or cell clusters, forming 1 small protrusion. (**E,K** and **L**) Outward protrusion of the lateral surface of the cell chain or cell cluster, forming several small protrusions.

Experimental observation indicated that the strains exhibited both budding and binary fission at the same time during the cell growth process, and subsequent data analysis confirmed that this phenomenon was not accidental or isolated. Binary fission is a simple form of bacterial cell division (Fig. 2A)^5^, whereas the most common mode of vegetative growth in yeast is asexual reproduction by budding^6, 7^. However, the budding phenomenon has never been observed in LAB.

**Figure 3:**
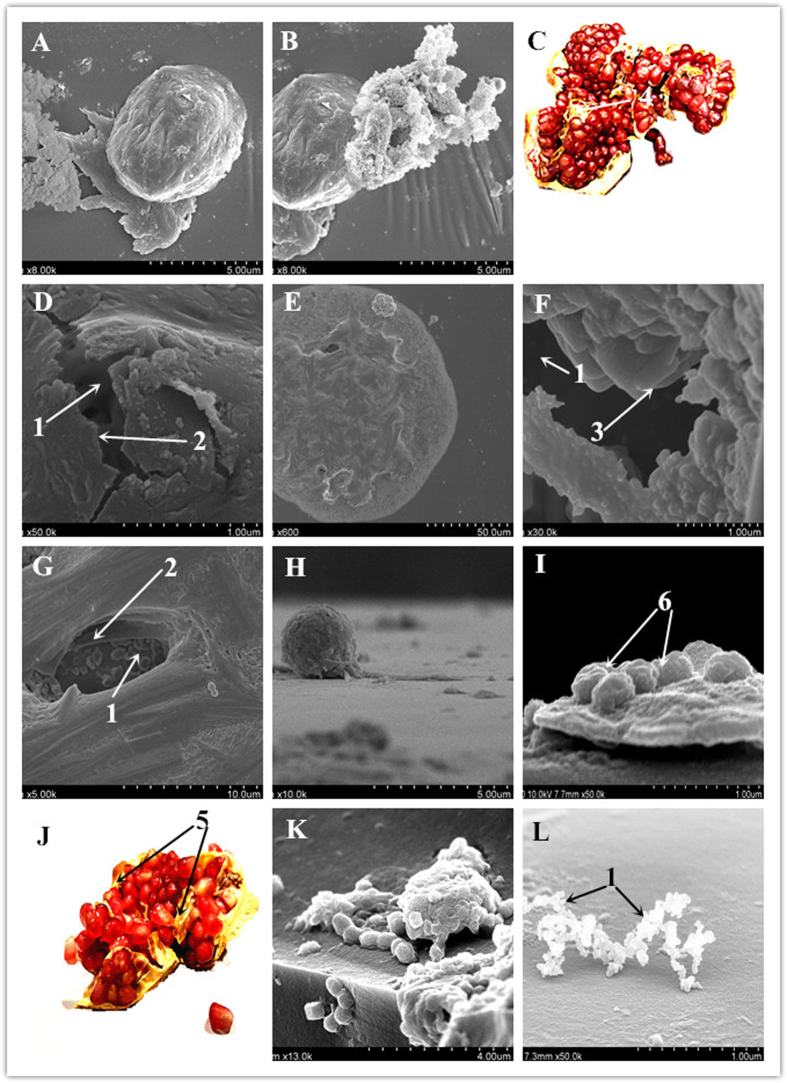
Structure and phenomenon of PSS. (**A**) A complete sphere of PSS. (**B**) Broken PSS. (**C**) Broken pomegranate. (**D**) Internal structure of PSS. (**E**) A complete cell of the PPS. (**F**) Close-up of the hole in the upper part of the cell; the lower part of the gap, torn membrane, and internal structure are clearly visible. (**G**) One diplococcus at close range, showing the internal stent and debris, as well as the internal fragment and structure. (**H–I,K**) Stereo or planar images illustrating the complete or incomplete rupture of PSS and the different sizes of debris, secretions, or ejecta. (**J**) Structure of a pomegranate fruit. (**L**) Various shed internal clusters or grape-like cells.

In the present study, the cell budding mode accounted for 4.7% of the measured data (Table 2), markedly different from binary fission during the growth process. Overall, 60.5% of the cells exhibited a size between 200 nm and 600 nm. The cell shapes were more complex but exhibited some common and changing structural characteristics, which can be summarized in 3 ways. First, the centre of the surface of spherical cells protrudes outward, forming 1 (Fig. 2A–C) or several small protrusions. Then, the size of the protrusion increases, and the protrusions separate from the mother cell after growing to a normal size, forming independent cells. When the bacterial cells are in the lag phase of individual development or when the cells are under suitable growth conditions, the bud continues to grow until it separates from the parent cell, forming a new cell. Yeong^8^ noted that at the end of nuclear division in budding yeast, acto-myosin ring contraction and cytokinesis occur between mother and daughter cells. This is followed by cell separation, after which mother and daughter cells go their separate ways. This is very similar to observations in Fig. 1E–1. The successive growth and changes in BBF are shown in Figs 1 A–1, 2, and 3.

**Table 2:** The data measured from the images.

	Number of measured data.	Select the measurement picture	Percentage of all pictures	N6	N7	N11
BBF	291	83	4.7%	56.6%	28.3%	15.1%
PSS	793	238	13.5%	66.0%	20.3%	13.7%
Spherical	445	251	14.3%	/	/	/
Chains	1622	446	25.4%	/	/	/

Strain code: N6, SX-1a; N7, SD-1g; N11, SD-2a.

Second, the lateral surface of the cell chain (i.e. the normal position of binary fission) protrudes outward, forming 1 (Fig. 2D and F) or several small protrusions (Fig. 2G). Then, as in the first type, the size of the protrusion increases, and the protrusions separate from the mother cell after growing to a normal size, resulting in independent cells. In the stationary phase and decline phase, the individual cells under adverse growth conditions (such as aging) will appear symmetrical or demonstrate asymmetric budding (multilateral budding)^9^. Reid *et al*.^10^ and Lipke *et al*.^11^ speculated that changes in the major wall polysaccharide and the plasma membrane might cause these morphological changes.

**Figure 4:**
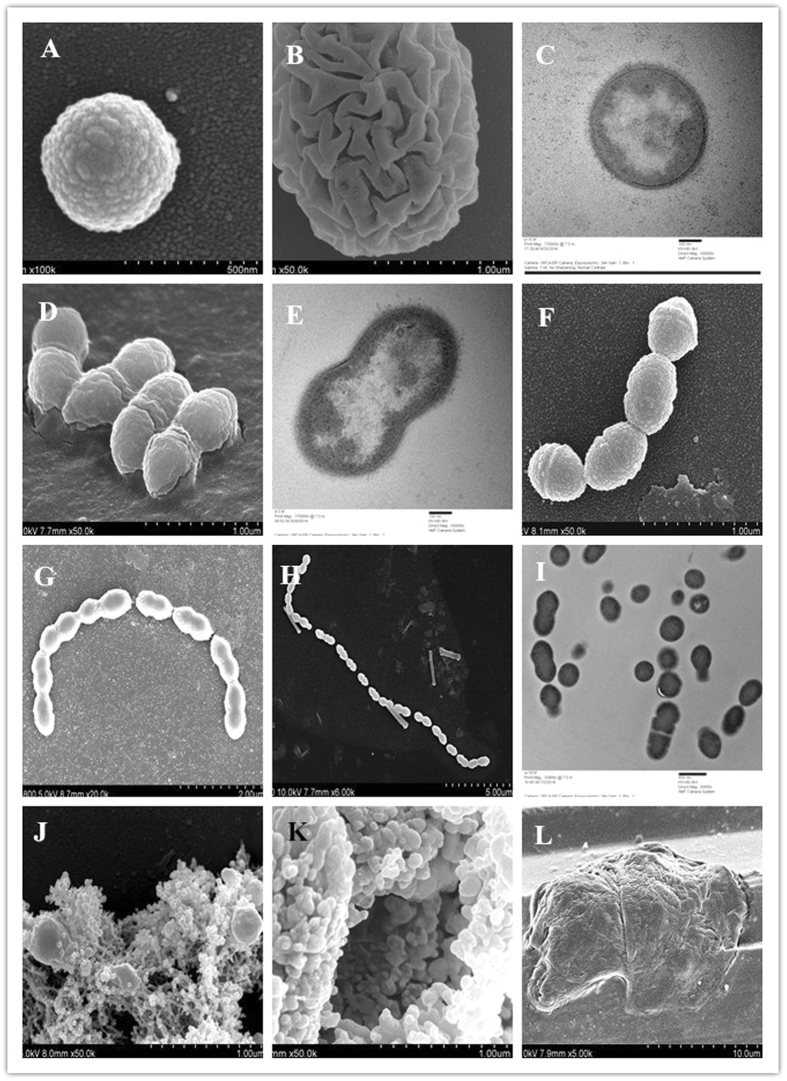
Indications of SCP. (**A**) Spherical growth process. (**B**) Protrusions or indentations of the cell. (**C**) The cell wall with visible cell membrane integrity, intracellular organelles, and a visible nucleoid. (**D,E** (TEM), and **F**) Binary cell fission; chains with 2–8 spherical parts could be observed. (**G**) Binary cell fission exhibiting a longer chain appearance (16 globular parts). (**H**) During cell division, the membrane extension was clearly visible between the spherical cells. (**I**) Short chains of cells as observed by TEM. (**J–L**) The rupture or eruption of the PSS.

Finally, in the third type, an outward protrusion (Fig. 2H–J) or several small protrusions (Fig. 2E,K, and L) occur on the lateral surface of cell chains or cell clusters, although the position of this kind of protrusion appears to be random (e.g. axial asymmetry of cell division). Then, the protrusions increase in size, perhaps separating from the mother cell after growing to the normal size and becoming independent cells. Notably, bacterial budding was observed during all the cell growth periods. Under unfavourable growth conditions, cells appear symmetrical or demonstrate asymmetric budding (multiple buds). These successive growth patterns and changes are shown in Figs 2 E– 4, I–5, and L–6. Anna *et al*.^12^ noted that under unfavourable temperature, the cell wall composition differs from that under normal temperature, often becoming endospore-forming. Similar results have been found in *Proteobacteria* ^13–16^. Some research^17–19^ suggests that when the oxygen level is not optimum, the cells will show symmetrical or asymmetrical budding. This might be ascribed to oxidative stress, leading to oxidative DNA damage in cells. However, LAB strains that exhibit improved aerobic stability^20–25^ have indicated that when nutrients are limiting (nutrient starvation), cells show multilateral budding.

The discovery of BBF has led us to consider a new possibility in the traditional theory of cell ontogeny in LAB. First, we speculate that *O*. *oeni* cells may display a new rapid proliferation process in the growth process, which is particularly evident in sample N6 (56.6%) (Table 2). A kind of stress response may also exist, caused by occasional changes in the microscopic environment of cell growth. Given that during these changes, the experimental strain N11 exhibited little change (15.1%) and showed relatively stable performance (homeostasis), this strain was screened out^26^.

### Pomegranate-shaped structure

The observation of a PSS represents a new finding. This feature occurred mainly in the stationary phase and the decline phase. In addition, during an FESEM observation, the view of the huge spherical strain suddenly disappeared; however, when we reduced the resolution to search for it, the cell was accidentally hit by a high-energy electron beam, causing a displacement. Therefore, we had the opportunity to observe the unique internal structure of the cell. Figure 3A shows the complete sphere; the broken PSS is shown in Fig. 3B, with a broken pomegranate fruit shown in Fig. 3C for comparison. At a close range, the gap and a rolled dough-like substance showing many cocci can be seen, the internal structure being clearly visible. Figure 3D shows the cell gap of the upper part at a close range. The ruptured membrane, internal opening, and other structures are clearly visible. The conformation of the bacteria from the external ‘skin’ or membrane to the internal structure, including separate brackets and the separation between the huge clusters of cells, closely resembles the structure of a pomegranate (Fig. 3J); thus, we termed this unique structure the PSS.

The individual cells (65.2%) were between 200 and 600 nm in size, presenting a clear PSS with a diameter above 2,000 nm. Figure 3E shows a complete cell; Fig. 3F shows the lower part of the gap, with the torn membrane and the internal structure clearly visible. At the upper part of the cell, a hole could be observed. At a close range, a diplococcus was visible (Fig. 3G), showing the internal stent and debris, as well as the internal fragments and structures. During this period, the cell morphology was observed to be very complex. In stereo or planar images, complete or incomplete rupture of the PSS could be observed (Fig. 3H). The different sizes of debris, secretions, or ejecta can be seen, and internal chains and globular and shedding clusters or cells with a grape-like appearance are visible (Fig. 3I,K, and L).

From these observations, that the cells exhibit some common structural as well as changing characteristics, we suggest that a single complete PSS might comprise 3 parts, described as follows. (1) PSS internal structure and changes: A scaffold and septum exhibiting different shapes and distributions during cell growth might be present. Initially, the encapsulation of the cells in the scaffolds and septum supports and protects the cells. With the continuous growth of cells, the scaffolds and septum eventually become thinner by compression from the cells. These successive changes are shown in Fig. 3D-L, G-2, F-3, C-4, J-5, and I-6 ^27^. (2) Cells and cell clusters: The interior of the pomegranate fruit consists of individual seeds separated into compartments by membranous walls and white spongy tissue^28^. *O*. *oeni* cells showed different sizes and morphologies during different growth stages, and gradually grew and expanded from the smaller, individual cells in the early stages, and adjacent cells formed clusters of cells in the septum (Fig. 3 F- 1, G-1, L-1). (3) Changes in external membranes and their effects: The cells were found to be enclosed within an external covering of a soft, thin membrane (Figs 3 D– 2), akin to the individual seeds of the pomegranate fruit^29^. However, under the influence of some unfavourable growth conditions (e.g., temperature, moisture, aging, hypoxia, etc.)^30^, the thin outer membrane becomes tough and thickened. Meanwhile, the internal cell wall also changes accordingly. This may affect the growth of cells. Cells are confined in a flexible double layer of the shell, with the internal cells continuing to grow, increase, and split, However the shell (capsule) envelops the internal changes until the shell cannot stand the increasing internal growth pressure, resulting in the rupture of the PSS shell (Fig. 3B,C,J and L).

Therefore, we speculate that PSS first emerged as a form of bacterial growth arising from a stress response to the changes in the microenvironment, used to protect and complete the growth and proliferation of strains. It is also possible that the structure reflects a type of individual development of the cells that has not been identified yet. As in the BBF analysis data, sample N6 was prominent with respect to the PSS characteristics (Table 2), whereas sample N11 had least evident features. Sample N6 accounted for 66.0% of observed PSS; N7, 20.3%; and N11, 13.7%. Sample N11 was relatively stable (patented strain).

### Symbiosis and cyclical phenomena

FESEM showed that the cells were spherical or oval, with 70.6% of the cell diameters being between 31.8 and 600 nm. The initial growth surface was smooth or with homogeneous peptidoglycan particles visible as uniform fine points; the particle diameter was between 8.94 and 12.9 nm, averaging 10.9 nm. Folds resulting from the growth process can be seen in Fig. 4A; the protrusions can be clearly observed in the image of the 2 layers of the cell chains and sleeve membrane in Fig. 1F, and the recessed regions are highlighted in Fig. 4B. At the decline phase, many cells were ruptured, with visible cracks and holes. Homogeneous particles of peptidoglycan can be seen in the background upon visible observation. TEM observations of the cell wall, cell membrane integrity, and intracellular organelles with a visible nucleoid (Fig. 4C) are consistent with the FESEM observations.

Over the major portion of the experiment, numerous types chains were observed. FESEM showed 93.2% of the diameters of the spherical part of the cell to be between 300 and 600 nm, and the cell surfaces were smooth, with homogenous peptidoglycan particles presenting as uniform fine points (>86%) until the decline phase. A larger percentage of cells exhibited homogeneous peptidoglycan particles in the background upon visual observation (>89%). At this stage, the cell division consisted mostly of binary fission. Most of the chains (>70%) contained 2–8 spherical parts (Fig. 4D–F), although some were longer (>10 globular parts) (Fig. 4G). During cell division, the membrane extension was clearly visible between the spherical cells (Fig. 4H). By TEM, short chains of cells (2–4) were clearly visible, whereas long chains (such as those with >4 spherical cells) could not easily be observed (Fig. 4I). Following this stage, the PSS appeared.

This scheme appears to proceed in the following order: a spherical cell converting into a cell chain, its expansion into a PSS, rupture of the PSS (Fig. 4J and K), and then, yet another cycle. Therefore, the cell growth process is cyclical, embodying the continuity of life. In all the images, we determined that the background consistently exhibited a uniform peptidoglycan particle diameter (8.94–13.9 nm, averaging approximately 11.4 nm), which provided further evidence that the observed variability in morphologies associated with the phenomena of BBF, PSS, and SCP were not artefacts. Moreover, the co-existence of synergistic phenomena during the cell growth process could also be observed, as we found that a single image could illustrate the end of a cell’s life after the start of a new life (Fig. 4L).

The cells of *O*. *oeni* are spherical to elliptic, usually in paired and chain-like arrangement^31^. However, this ‘living together’ phenomenon has never been described in terms of this LAB. Here, we considered the established concept of ‘symbiosis’ as being more suitable to explain it. The term is derived from Greek συμβίωσις (meaning living together), from σύν ‘together’ and βίωσις ‘living’^32^. We chose this term because it refers to any type of close and long-term biological interaction between 2 different species, be it mutualistic, commensal, or parasitic. In 1879, Anton de Bary defined symbiosis as ‘the living together of unlike organisms.’ Mutualistic flora are known to play a crucial role in digestion^33–35^. Symbiotic bacteria oxidize hydrogen sulphide or methane, which the host supplies to them; such a relationship is called obligate mutualism^36^. Temperature and relative humidity conditions influence growth and death rates of various microorganisms^37^, and the composition and activity of the gut microbiota codeveloping with the host from birth is subject to a complex interplay that depends on the host genome, nutrition, and lifestyle. Numerous species of gut microbiota are involved in the regulation of multiple host metabolic pathways^38^. Microbiota are very closely linked with many parameters of host biology in both health and disease^39^. Einar *et al*.^40^ set up a good question: Does the gradual development of the gastrointestinal tract, from the larval stage to the adult stage, affect infection? This question reflects the impact of changes in time and space, demonstrating the mutual impacts of microbiota as complex and spectacular.

In conclusion, we identified 3 structural phenomena in the cell growth process of *O*. *oeni* as inferred using over 2,000 FESEM and TEM images. We believe that there is a certain relationship whereby BBF occurs because of the effects of cells on the environment, rather than the other way around. Alternatively, the cells cause a particular microenvironment and respond to it. First, the existence of BBF in the experimental strains is not an accidental and isolated phenomenon in the growth process. Second, the morphologies of the strains at different growth stages are mainly spherical, chain-like, and PSS, with the latter structure revealing the complex growth process of the cell. Third, the different growth periods of the experimental strains exhibit SCP features. We consider that a prior lack of recognition of this complexity might underlie the constant changes in the classification of the wine LAB *O*. *oeni*.

These experiments were conducted under identical temperature conditions. In future studies, we hope to modify various factors (e.g. temperature, pH value, alcohol percentage), or add exogenous biological or chemical agents to examine the effects of autologous and environmental factors on these phenomena and the promotion and inhibition of cell growth to deepen our understanding of the structure and function of the cells. We intend to compare the bacteria exhibiting BBF and PSS with the common bacteria *Streptococcus* and *Staphylococcus aureus* to determine whether they exhibit similar morphologies or have similar genes controlling such phenotypes. This would also address the detailed processes and mechanisms of the formation and changes in BBF and PSS, and allow us to explore the relationship between and mechanism of the initiation and termination of MLF by the PSS.

## Materials and Methods

### Bacterial strains and growth conditions

The *O*. *oeni* strains were obtained from our own collection (College of Enology, Northwest A&F University, Yangling, Shaanxi, China). These 22 strains (Table 3) were previously isolated from different provinces in China and were properly identified. *O*. *oeni* 31DH (*O*. *oeni* CICC 6066) was provided by the China Center of Industrial Culture Collection (CICC)^41^. The preserved strains were activated in FT80 liquid culture medium (pH 4.5, 25 °C)^42^. On the sixth day, 200 μL bacterial liquid from sealed cultures was collected and purified in a 9-cm petri dish^43^ (FT80 solid culture medium, pH 4.5, 25 °C). Table 4 shows the time of sampling for each strain on the sixth day of purification culture. For sampling, a colony was dissolved in 0.1 mL distilled water on a sterilized round coverslip (6 × 6 mm).

**Table 3:** Sampling codes.

Code strain preserved	1	2	3	4	5	6	7	8	9	10	11	12
Code strain	HB-1a	SD-1c	SD-2gh	SD-1a	SD-2kj	SX-1a	SD-1g	SD-2h	HB-2b	SD-2d	SD-2a	SX-1b
Code sampling	A	B	C	D	E	F	G	H	I	J	K	L
Code strain preserved	13	14	15	16	17	18	19	20	21	22	23	
Code strain	SD-2gf	HB-1b	SD-1b	HB-1c	SD-2ji	SD-1f	31-DH	SD-2b	SD-1e	SD-2ee	SD-1d	
Code sampling	M	N	O	P	Q	R	S	T	U	V	W	

Strain code: HB, Hebei province; SD, Shandong province; SX, Shaanxi province (China).

**Table 4:** Code and interval time of sampling during the culture process (for FESEM).

Sampling code	1–5	6–11	12–23	24–27	28–30	31	32
Interval time of sampling (h)	6	2	1	6	18	240	480

Strain code: N6, SX-1a; N7, SD-1g; N11, SD-2a.

### Sample treatment and observation

#### Gram staining (Gram’s method)

Samples were processed as described previously^44^. A DP70 digital camera (Olympus, Japan) was used for image capture and to observe Gram-positive staining.

#### Field-emission scanning electron microscopy

The experimental sample and 3 replicates were washed in distilled water, sterilized, and analysed using electron microscopy. The samples were fixed with 2.5% glutaraldehyde in 0.1 M sodium phosphate buffer (pH 7.2), for 2 h at 4 °C. After 3 washes with 0.1 M sodium phosphate buffer (pH 7.2), the samples were dehydrated in a graded series of ethanol, treated with isoamyl acetate twice, and dried in a critical-point drying machine (Emitech K850; Quorum) as described (Method 1)^45^ or naturally dried (Method 2). The dried samples were placed at the bottom of 2-mL conical Eppendorf tubes, with 1% Osmic acid fumigation fixation for 4 h (Method 3) or 2 h (Method 4, the finally selected method). After metal spraying, samples were analysed in an S4800 field-emission scanning electron microscope (Hitachi). Control and sample groups were investigated.

#### Transmission electron microscopy

The sample was dried and placed at the bottom of a 2-mL conical Eppendorf tube and centrifuged for 10 min (6,000 × *g*). After supernatant removal, the sample was dropped into rapid solidifying agar, mixed, cooled, removed, and sliced.

The experimental sample and 1 replicate were washed with distilled water, and fixed with 2.5% glutaraldehyde in 0.1 M sodium phosphate buffer (pH 7.2) for 6 h at 4 °C. After the segments were washed 5 times with 0.1 M sodium phosphate buffer (pH 7.2), they were post-fixed in 1% Osmic acid in 0.1 M sodium phosphate buffer (pH 7.2) for 2.5 h (Method 1)^45^, 2 h (Method 2), or 4 h (Method 3, the final selected method). They were then washed with 0.1 M sodium phosphate buffer (pH 7.2), dehydrated in a graded series of ethanol baths, and embedded in Epon-812. Ultrathin sections were stained with uranyl acetate and lead citrate and examined using an HT7700 transmission electron microscope (Hitachi, Ltd.).
